# Validation of EGSYS Score in Prediction of Cardiogenic Syncope

**DOI:** 10.1155/2015/515370

**Published:** 2015-11-16

**Authors:** Hamid Kariman, Sepideh Harati, Saeed Safari, Alireza Baratloo, Mehdi Pishgahi

**Affiliations:** ^1^Emergency Department, Imam Hossein Hospital, Shahid Beheshti University of Medical Sciences, Tehran 19899 34148, Iran; ^2^Emergency Department, Shohadaye Tajrish Hospital, Shahid Beheshti University of Medical Sciences, Tehran 19899 34148, Iran; ^3^Cardiology Department, Shohadaye Tajrish Hospital, Shahid Beheshti University of Medical Sciences, Tehran 19899 34148, Iran

## Abstract

*Introduction*. Evaluation of Guidelines in Syncope Study (EGSYS) is designed to differentiate between cardiac and noncardiac causes of syncope. The present study aimed to evaluate the accuracy of this predictive model.* Methods*. In this prospective cross-sectional study, screening performance characteristics of EGSYS-U (univariate) and EGSYS-M (multivariate) in prediction of cardiac syncope were calculated for syncope patients who were referred to the emergency department (ED).* Results*. 198 patients with mean age of 59.26 ± 19.5 years were evaluated (62.3% male). 115 (58.4%) patients were diagnosed with cardiac syncope. Area under the ROC curve was 0.818 (95% CI: 0.75–0.87) for EGSYS-U and 0.805 (CI 95%: 0.74–0.86) for EGSYS-M (*p* = 0.53). Best cut-off point for both models was ≥3. Sensitivity and specificity were 86.08% (95% CI: 78.09–91.59) and 68.29% (95% CI: 56.97–77.86) for EGSYS-U and 91.30% (95% CI: 84.20–95.52) and 57.32% (95% CI: 45.92–68.02) for EGSYS-M, respectively.* Conclusion*. The results of this study demonstrated the acceptable accuracy of EGSYS score in predicting cardiogenic causes of syncope at the ≥3 cut-off point. It seems that using this model in daily practice can help physicians select at risk patients and properly triage them.

## 1. Introduction

Syncope is a transient loss of consciousness and postural tone, which is responsible for about 5% of emergency department (ED) referrals [1]. This symptom has a wide range of causes from nonserious to potentially fatal [2–8]. Accurate cause prediction and choosing the proper approach to these patients have been historical challenges for physicians [9]. Causes of syncope can be broadly divided into cardiac and noncardiac. Usually, patients with cardiogenic syncope have a higher mortality rate regardless of their age [2]. Clinical and electrocardiography (ECG) findings can be helpful in the classification of syncope with cardiac origin [1, 10]. In some developed countries, syncope units are responsible for the management of these patients, which have efficiently reduced unnecessary hospitalization [1, 4, 5]. Yet, in most countries, these units cannot be established and simpler strategies are used for triage of these patients. One of these strategies is using clinical risk stratification rules such as San Francisco syncope rule, short-term prognosis of syncope (STePS), and Evaluation of Guidelines in Syncope Study (EGSYS) [5, 9, 11–18]. Most of these models predict the patients' outcome, while EGSYS has been designed to differentiate between cardiac and noncardiac causes of syncope. In this model, patients are given a score based on the presence or absence of risk factors such as abnormal ECG findings, palpitation, precipitating or predisposing factors, and autonomic prodromes. Higher scores are in favor of cardiac causes. Previous studies have introduced EGSYS as an efficient and sensitive tool in the prediction of cardiogenic syncope [3, 12]. In a cohort study by Del Rosso et al. sensitivity and specificity of this model were reported to be 92% and 69%, respectively [12]. Based on the above-mentioned points, the present study was designed to validate EGSYS scores in the differentiation of cardiac and noncardiac causes of syncope.

## 2. Methods

This prospective cross-sectional validation study was carried out on syncope patients who were referred to ED of three University Hospitals, Tehran, Iran, from March 2012 to March 2013. This study was aimed to evaluate the accuracy of EGSYS scores in the prediction of cardiogenic syncope. Protocol of the present study was approved by the ethics committee of Shahid Beheshti University of Medical Sciences and the researchers adhered to the principles of Helsinki Declaration. Informed written consent was obtained from all of the studied patients. All patients >18 years old, who were referred within 24 hours of symptom initiation, were included. The subjects were enrolled using nonprobability consecutive sampling by Sepideh Harati during her routine 12-hour residency shifts (day or night) under direct supervision of attending physician on duty. Patients, who did not give their consent or the origin of their syncope was unknown, were excluded. Initially, enrolled patients were physically examined by the senior emergency medicine resident (Sepideh Harati) and their demographic data (age, sex) as well as EGSYS risk factors (Table 2) were recorded using a predesigned checklist [1]. Then, the patients were followed until their final cause of syncope (cardiac or noncardiac) was determined. For patients whose cause of syncope was clear during their hospitalization, data were recorded from patients' files. While cases, that were discharged before confirmation of diagnosis and scheduled for additional outpatient tests such as electrophysiological study, which was not available in the studied centers, were followed using telephone call with patients or their in charge family physicians. Therefore, follow-up period varied from one week to one month in some cases. Next, univariate and multivariate EGSYS scores were calculated for all the patients. The patients' ECGs were interpreted by an independent cardiologist who was blind to the clinical status and diagnosis of the patient. Final decision on the probable cardiac cause of syncope was made based on the results of echocardiography, stress testing, prolonged Holter ECG monitoring, and electrophysiological study. In cases of normal cardiac evaluations, probable neurologic causes were ruled out using tilt testing, brain imaging, and carotid massage [7]. The origin was reported as unknown if the above-mentioned evaluations were normal.

### 2.1. Calculation of EGSYS Score

At first, the presence of risk factors listed in Table 2 was evaluated for each patient. Then, the sum of the scores was calculated for each patient. Those with a total score ≥3 were considered at risk for presence of cardiac cause.

### 2.2. Definitions

Since the present research aimed to validate the study by Del Rosso et al. [12], all terms and definitions were based on the original derivation study.

- ECG abnormality was considered as the presence of one or more of the following abnormalities: bradycardia (<40 beat/minute), ST changes (>1 mm elevation or depression), QT prolongation (440 ms), ventricular tachycardia, atrioventricular block (second or third degree), sick sinus syndrome, ventricular and rapid paroxysmal supraventricular arrhythmias, sinus pauses, and pace malfunction.
- Predisposing or precipitating factors were considered as the presence of one or more of the following abnormalities: warm place, crowded place, prolonged standing, overtiring, postprandial period, neck turning, syncope while sitting, supine, or upright, syncope during effort, and syncope after effort.
- Prodromal symptoms and signs were considered as the presence of one or more of the following abnormalities: blurred vision, lightheadedness, diaphoresis, palpitations, nausea, vomiting, abdominal discomfort, weakness, feeling cold, feeling warm, tremors, yawn, pallor, redness, or cyanosis.

### 2.3. Statistical Analysis

Considering 90% sensitivity, 5% desired precision, and 95% confidence interval (CI), the minimum sample size was calculated to be 138 cases. Screening performance characteristics of EGSYS score (sensitivity, specificity, positive and negative predictive values, and positive and negative likelihood ratios) in prediction of cardiogenic syncope were calculated using SPSS version 21.0. Receiver operating characteristic (ROC) and area under the curve with 95% CI were used to find the best cut-off point and accuracy of the model. *p* < 0.05 was considered significant.

## 3. Results

**Figure 1:**
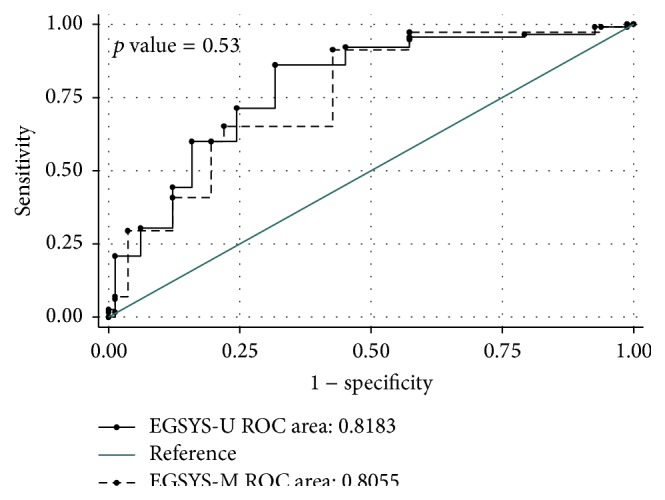
Comparative receiver operative characteristic (ROC) curve of univariate EGSYS (EGSYS-U) and multivariate EGSYS (EGSYS-M).

206 patients were evaluated; however, 8 cases with mean age of 50.0 ± 22.74 (75.0% male) were excluded due to unknown origin of syncope. No cases of miss or death happened during the patients' follow-up period. The EGSYS-M score of all excluded patients were ≤2. Finally, 198 patients with mean age of 59.26 ± 19.5 years (range: 13–98) were enrolled in the study (62.3% male).  Table 1 shows baseline characteristics of the participants. Frequency of EGSYS risk factors is shown in Table 2. 115 (58.4%) patients were diagnosed with cardiac and 83 (41.6%) with noncardiac origin. The comparison of EGSYS risk factors between cardiac and noncardiac syncope can be found in Table 3. Mean EGSYS-U and EGSYS-M scores were 3.57 ± 3.05 (minimum −4 and maximum 10) and 3.73 ± 2.68 (minimum −2 and maximum 10), respectively. Mean EGSYS-M and EGSYS-U scores for cardiac and noncardiac causes were 4.9 ± 2.1 and 2.11 ± 2.4 (*p* < 0.001) and 5 ± 2.5 and 1.6 ± 2.6 (*p* < 0.001), respectively.  Figure 1 shows the comparative ROC curve of the 2 mentioned models. Area under the ROC curve was 0.818 (95% CI: 0.75–0.87) for EGSYS-U and 0.805 (CI 95%: 0.74–0.86) for EGSYS-M (*p* = 0.53). Best cut-off point for both models was estimated to be ≥3.  Table 4 summarizes screening performance characteristics of the 2 mentioned models. At ≥3 cut-off point, sensitivity and specificity were 86.08% (95% CI: 78.09–91.59) and 68.29% (95% CI: 56.97–77.86) for EGSYS-U and 91.30% (95% CI: 84.20–95.52) and 57.32% (95% CI: 45.92–68.02) for EGSYS-M, respectively.

## 4. Discussion

Based on the results of this study, EGSYS score at ≥3 cut-off point shows acceptable sensitivity for screening the patients with cardiogenic syncope. No significant difference was seen between the univariate and multivariate models in this regard. Mean EGSYS score of the patients with cardiac causes was significantly higher (about twice as much). The value of clinical findings and characteristics of syncope in prediction of patient outcomes have been evaluated in different studied [12, 18, 19]. In addition, various clinical decision rules have been designed and validated for this purpose [5, 9, 11–18]. Among them, EGSYS claims to be able to differentiate between cardiac causes and noncardiac causes of syncope. A study on 153 patients in 2008 showed that EGSYS is an efficient tool to distinguish cardiogenic syncope, reducing unnecessary hospitalization and improving ED management [18]. A study by Del Rosso et al. in 2008 also demonstrated that EGSYS has high sensitivity in predicting cardiac causes of syncope [12]. One-month and 2-year outcome assessment of 465 syncope patients in 2010 (EGSYS-2 study) demonstrated an increased mortality rate in patients with higher EGSYS scores [19]. The results of one cohort study estimated the sensitivity of EGSYS in predicting mortality and bad outcome to be 80% and 56%, respectively [20]. In the present study, best cut-off point for differentiating cardiac causes of syncope for both univariate and multivariate models was ≥3, which is in line with the results of the previous studies [12, 18]. Area under the ROC curve in the present study for both models was above 80%, which represents a good level of accuracy. Among the EGSYS risk factors, highest odds ratios belonged to ECG abnormality (11.27), palpitation (7.48), and blurred vision (5.12) which were lower than these values in the Del Rosso et al. study, except for ECG abnormality. Regarding the high percentage of cardiogenic causes in comparison to previous studies [12, 21], two points should be mentioned: (1) the sampling is not census and (2) the end point for data gathering was reaching at least 138 cases of cardiac syncope; therefore, this finding is not relevant to prevalence of cardiogenic syncope.

*Limitations*. Sample size for the present study was estimated to be 138 cases of cardiogenic syncope, but, in the end, the study was done with 115 cases. Since the power of this study was 99.99% based on comparing the mean EGSYS score in cardiac and noncardiac groups, the aforementioned deficiency has no effect on the results of study. On the other hand, it should be considered that some syncope patients are never referred to ED due to rapid improvement of clinical status, most of which have probably had benign noncardiac causes. This can shift the composition of the participants in favor of cardiogenic causes and affect the results. Therefore, we need comprehensive multicentric studies to be able to generalize the findings. In addition, evaluating the short- and long-term outcome of the patients can aid in making more adequate and accurate decisions.

## 5. Conclusion

The results of this study demonstrated the acceptable accuracy of EGSYS score in predicting cardiogenic causes of syncope at the ≥3 cut-off point. There was no significant difference between the univariate and multivariate models in this regard. It seems that using this prediction model in daily practice can help physicians in the selection of at risk patients and proper triage of them for further evaluations.

## Key Elements

One can find the following.

1. EGSYS score can predict cardiogenic causes of syncope with acceptable accuracy.
2. The best cut point of model for this purpose is score ≥3.
3. There is no significant difference between the univariate and multivariate models.
4. It could be helpful in triage of syncope patients.

## Figures and Tables

**Table 1:** Baseline characteristics of the studied patients.

Variable	Values
Systolic blood pressure (mmHg)	
Maximum	123.87 ± 26.72
Minimum	74.87 ± 12.06
Pulse rate (per minute)	75.87 ± 18.75
O_2_ saturation (%)	95.57 ± 4.16
Hemoglobin (mg/dL)	12.63 ± 1.5
Tilt positive	11 (5.6)
ECG abnormality	71 (36.2)
T-changes (elevation, depression)	44 (22.6)
ST changes (>1 mm)	14 (7.2)
QT >440 milliseconds	21 (14.3)
Atrioventricular block	3 (1.6)
QRS >120 milliseconds	32 (25.4)
History of syncope	
No	142 (73.6)
Yes	46 (23.8)
Occurrence of previous syncope	
≤1 month	42 (91.3)
<1 month	4 (8.7)
<1 week	32 (69.6)
Position	
Supine	19 (9.9)
Upright	116 (60.4)
Sitting	33 (17.2)
Activity during syncope	
Effort	68 (35.8)
Resting	54 (28.4)

Values were presented as mean ± standard deviation or number and percentage.

**Table 2:** Frequency of EGSYS risk factors in the studied patients.

Risk factors	Number (%)	OR^3^ (95% CI^4^)	*p* value	Score^#^
EGSYS-U^1^				
Abnormal ECG^*∗*^/cardiopathy	134 (67.3)	11.27 (5.42–23.01)	<0.001	3
Palpitations/dyspnea	59 (30.1)	7.48 (3.3–16.96)	<0.001	3
Syncope in supine position/effort syncope	68 (34.2)	1.37 (0.74–2.40)	0.315	2
Age >64 years	87 (45.1)	4.54 (2.24–8.53)	<0.001	1
No precipitating and predisposing factors	128 (65.6)	0.84 (0.46–1.53)	0.576	1
No prodromes^*∗∗*^	46 (23.6)	3.84 (1.73–8.52)	0.001	1
Blurred vision	40 (20.7)	5.12 (2.37–11.06)	<0.001	−1
Neurovegetative signs during recovery phase	68 (35.2)	2.19 (1.20–4.01)	0.010	−1
Precipitating and predisposing factors^*∗∗∗*^	67 (34.4)	0.84 (0.46–1.53)	0.575	−2
Autonomic prodromes	67 (34.2)	0.13 (0.02–1.05)	0.055	−2
EGSYS–M^2^				
Abnormal ECG/cardiopathy	134 (67.3)	11.27 (5.42–23.01)	<0.001	3
Palpitations/dyspnea	59 (30.1)	7.48 (3.3–16.96)	<0.001	4
Effort syncope	51 (26.4)	0.94 (0.49–1.79)	0.844	3
Syncope in supine position	9 (9.8)	4.28 (1.20–15.25)	0.016	2
Autonomic prodromes	67 (34.2)	0.13 (0.02–1.05)	0.025	−1
Precipitating and predisposing factors	67 (34.4)	0.84 (0.46–1.53)	0.575	−1

^*∗*^Presence of one or more ECG abnormality listed in Definitions.

^*∗∗*^Defined in Methods.

^*∗∗∗*^Defined in Methods.

^#^Total score ≥3 indicates patients at risk for presence of cardiac cause.

^1^EGSYS-U: Evaluation of Guidelines in Syncope Study-Univariate.

^2^EGSYS-M: Evaluation of Guidelines in Syncope Study-Multivariate.

^3^OR: odds ratio.

^4^CI: confidence interval.

**Table 3:** The comparison of EGSYS risk factors between cardiac and noncardiac causes of syncope.

EGSYS^*∗*^	Cause of syncope		*p* value
	Noncardiac *N* (%)	Cardiac *N* (%)	
Abnormal ECG/cardiopathy	32 (24.1)	101 (75.9)	0.010
Palpitations/dyspnea	8 (13.6)	51 (86.4)	<0.001
Syncope in supine position/effort syncope	25 (36.8)	43 (63.2)	0.315
Age >64 years	20 (23)	67 (77)	<0.001
No precipitating and predisposing factors	55 (43)	73 (57)	0.575
No prodromes	9 (19.6)	37 (80.4)	≤0.001
Blurred vision	29 (72.5)	11 (27.5)	<0.001
Neurovegetative signs during recovery phase	37 (54.4)	31 (45.6)	0.010
Precipitating and predisposing factors	26 (38.8)	41 (61.2)	0.575
Neurovegetative prodromes	1 (9.1)	10 (90.1)	0.025

^**∗**^Evaluation of Guidelines in Syncope Study-Univariate.

**Table 4:** Screening performance characteristics of EGSYS-U^1^ and EGSYS-M^2^ in prediction of cardiac causes of syncope (cut-off point ≥3).

Screening performance characteristics	EGSYS-U (95% CI^3^)	EGSYS-M (95% CI)
Sensitivity	86.08 (78.09–91.59)	91.30 (84.20–95.52)
Specificity	68.29 (56.97–77.86)	57.32 (45.92–68.02)
Positive predictive value	79.20 (70.83–85.73)	57.00 (66.84–81.75)
Negative predictive value	77.78 (66.15–86.39)	82.46 (69.64–90.82)
Positive likelihood ratio	3.81 (2.67–5.42)	3.00 (2.22–4.06)
Negative likelihood ratio	0.29 (0.18–0.44)	0.21 (0.12–0.38)

^1^EGSYS-U: Evaluation of Guidelines in Syncope Study-Univariate.

^2^EGSYS-M: Evaluation of Guidelines in Syncope Study-Multivariate.

^3^CI: confidence interval.
